# Mental Disorders Forecast Alzheimer’s Disease and Related Dementias in 1.7 Million New Zealanders

**DOI:** 10.1093/geroni/igab046.693

**Published:** 2021-12-17

**Authors:** Leah Richmond-Rakerd, Stephanie D'Souza, Barry Milne

**Affiliations:** 1 University of Michigan, Ann Arbor, Michigan, United States; 2 University of Auckland, Auckland, Auckland, New Zealand

## Abstract

Neurodegenerative conditions, including Alzheimer’s disease and related dementias (ADRD), have an outsized impact on disability and loss of independence in older adults. As such, there is a growing need to identify modifiable risk factors for ADRD at the population level. We conducted a nationwide administrative-register study to investigate mental disorders as a potential preventable risk factor for later-life ADRD. Data were drawn from the New Zealand Integrated Data Infrastructure, a collection of whole-of-population administrative data sources linked at the individual level by a common spine. We identified all individuals born in New Zealand between 1928-1967 and followed them for three decades (N = 1,711,386; observation period = 1988-2018; age at baseline = 21-60 years). Diagnoses of mental disorders were ascertained from public-hospital records. Diagnoses of ADRD were ascertained from public-hospital records, mortality records, and pharmaceutical records. Individuals with a mental disorder were at elevated risk for developing Alzheimer’s disease and related dementias relative to those without a mental disorder. This prospective association was evident in both men and women, across age, and after accounting for pre-existing physical diseases. If associations are causal, ameliorating mental disorders could extend population healthspan and reduce the societal burden of neurodegenerative diseases.

